# Basal Cell Carcinoma: Comprehensive Review with Emphasis on Scar Tissue Manifestation and Post-Vaccination Incidence

**DOI:** 10.3390/biomedicines12081769

**Published:** 2024-08-06

**Authors:** Klaudia Knecht-Gurwin, Aleksandra A. Stefaniak, Iwona Chlebicka, Jacek C. Szepietowski

**Affiliations:** Department of Dermatology, Venereology and Allergology, Wroclaw Medical University, Chałubińskiego 1, 50-368 Wrocław, Poland; klaudia.knecht@gmail.com (K.K.-G.); aleksandraannastefaniak@gmail.com (A.A.S.); iwona.chlebicka@umw.edu.pl (I.C.)

**Keywords:** basal cell carcinoma (BCC), scar tissue, vaccination, BCG vaccination

## Abstract

Basal cell carcinoma (BCC) arising within scar tissue is a rare but clinically significant phenomenon. This comprehensive review aims to provide a succinct overview of the current state of knowledge regarding the etiological factors, pathogenesis, clinical presentation, and management of BCC. This study constitutes a literature review pertaining to BCC, with a particular emphasis on BCC developing within scar tissue. It also provides a clinical case presentation of a patient who had developed BCC in a BCG post-vaccination scar and a review of analogous findings available in the existing literature. Despite the fact that an array of mechanisms play a role in injury-related BCC growth, the main mechanism remains ambiguous and yet to be elucidated. The review also includes a detailed description of the various therapeutic options available for BCC, ranging from surgical interventions to novel pharmacological treatments. By examining these intersections, the review seeks to elucidate the potential mechanisms, identify risk factors, and suggest considerations for clinical practice. The findings underscore the importance of vigilant dermatological assessment in patients with scar tissue and those recently vaccinated, aiming to improve early detection and optimize management strategies for BCC.

## 1. Introduction

Basal cell carcinoma (BCC) cases comprise approximately 80% of malignant skin neoplasms. In Europe, the incidence is allegedly estimated at 400–800/100,000. The BCC rate frequency epitomizes an instance of an inadequately reported disease whose prevalence is undoubtedly higher [1]. The epidemiological research indicates a 10% increase in incidence every ten years. Typically, the onset of the disease occurs between the sixth and eighth decade of life with a slight male predominance. However, an expanded prevalence has been registered among the younger population, mainly women, which stems from increased UV exposure to artificial sources of the sun or inappropriate sunbathing [1]. The risk factors for BCC development encompass phototypes I and II according to Fitzpatrick’s scale, excessive exposure to UV radiation (particularly UVB), alterations in immunosurveillance, radiation therapy, HIV and HPV infections. Furthermore, excessive sun exposure in childhood has a pivotal role in BCC development [1]. Increased risk of BCC occurs in patients with genetic syndromes such as xeroderma pigmentosum, epidermolysis bullosa, Ferguson-Smith syndrome, Rasmussen syndrome, Muir–Torre syndrome, Rombo syndrome, Bazex–Dupré–Christol syndrome, albinism, and Darier’s disease [2]. Moreover, historically, microinjuries, long-lasting scars, non-healing ulcerations, deep abrasions, surgical incisions, and persistent skin irritation were considered contributory factors for SCC, but increasingly, these factors are also recognized as contributing to BCC development [1,3].

A few studies shed some light on the pathogenesis of BCC in trauma tissue and post-vaccinated tissue. Scar lesion features such as elasticity and decreased vascular and atrophic adnexal structure have been considered to make the covering epithelium more inclined to damage and malignancy. Another theory claims that cytokines and growth factor imbalance in scar lesions is the crucial factor for malignancy development [1,2].

## 2. Etiology, Pathogenesis, and Clinical Observations: Investigating the Multifaceted Aspects of Basal Cell Carcinoma

BCC arises from a complex interplay of genetic mutations, environmental factors, and immunological responses. The pivotal abnormality in BCC development is the malignant activation of the sonic hedgehog (SHH) signaling pathway, tightly regulated in normal adult tissues, but which plays a critical role in orchestrating fundamental processes such as the growth and organization of complex multicellular embryos [4]. The aberrant activation of the SHH pathway occurs primarily due to genetic alterations in key genes involved in the pathway [4,5].

Approximately 85% of sporadic BCCs harbor mutations in SHH pathway genes, including Patch 1 (*PTCH1*) smoothened (*SMO*), suppressor of fused (*SUFU*), and *TP53* [6,7,8]. The activation of SHH pathway yields an enhancement of transcription factors, such as Gli1, Gli2 and Gli3 (glioma-associated oncogene homologue 1, 2 and 3) [8]. These mutations, involving C to T substitutions at dipyrimidine sites, amount to UV radiation [9]. Somatic *PTCH1* gene mutations are detected in about 85–90% of BCCs cases, whereas approximately 10% reside in activating alterations in *SMO*, an oncogene normally suppressed by *PTCH1* [10]. Additionally, a small fraction of BCCs stems from alterations in the Patch 2 (*PTCH2*) gene, a homolog of *PTCH1* and a component of the SHH pathway [11]. Moreover, even BCC cases without detectable genetic alterations in the SHH pathway show upregulation in Gli1 and Gli2, transcription factors downstream of the SHH pathway, indicating a potential dysregulation of other molecules that activate this signaling [12].

Furthermore, frequent somatic inactivating mutations in the *TP53* gene are observed in BCCs, with a frequency of approximately 50%. *TP53* is known to be implicated in the early onset of various cancer types, including BCC, where the loss of heterozygosity (LOH) of p53, the protein encoded by *TP53*, seems to be mutually exclusive with *PTCH1* [10,13]. The mouse model denoted that loss of *TP53* implied an enhancement of the activity of the SHH pathway due to *SMO* expression augmentation [14]. Because p53 plays a crucial part in keratinocyte senescence, its loss of function may promote BCC development [8,9,13].

Moreover, while the SHH pathway is the primary driver of BCC, other pathways may also contribute to its genesis. These include the Hippo-YAP pathway and the MYCN/FBXW7 pathway. In rare cases, mutations in the epidermal growth factor receptor (EGFR) pathway, the phosphatidylinositol 3-kinase (PI3K)/protein kinase B (AKT) pathway, and members of the protein kinase C (PKC) family have also been reported [15].

It is noteworthy that in BCC development, the immunological aspects also play a crucial role. The interaction between cellular components in the tumor microenvironment (TME) ultimately contributes to the promotion of tumor growth by suppressing the immune responses mediated by dendritic cells and T cells. Specifically, tumor-associated macrophages (TAMs) and cancer-associated fibroblasts (CAFs) induce the upregulation of regulatory T cells (Tregs) through the release of interleukin-10 (IL-10), transforming growth factor-beta (TGF-β), and C-C motif chemokine ligand 22 (CCL22) [16,17]. Tregs, in turn, produce IL-10 and TGF-β, which inhibit the immune functions of dendritic cells (DCs) and T cells, thereby preventing the destruction of BCC tumor cells [18]. Furthermore, TAMs also secrete interleukin-6 (IL-6), which promotes angiogenesis, providing support for tumor growth and development [19,20].

## 3. Basal Cell Carcinoma Arising upon Scar Tissue

Interestingly, BCC can develop in areas of scar tissue, wherein the immunological microenvironment plays a crucial role [21]. This phenomenon highlights how trauma or alterations in the skin, such as those from vaccinations, can create an environment conducive to tumor development. By examining these cases, we gain insight into the mechanisms by which chronic inflammation and immune modulation in scar tissue can contribute to the pathogenesis of BCC. Such insights are crucial for understanding the broader context of BCC development, emphasizing that BCC can arise not only due to genetic and environmental factors but also as a result of specific local conditions. The concept of a causal link between traumatic injury and skin cancer was initially put forth by Virchow, a German pathologist, in 1863, yet this notion has persistently remained a topic of ongoing debate [22]. Contemporary research has further explored this relationship, shedding light on the connection between traumatic injury and the emergence of BCC, ultimately concluding that injuries can be regarded as an additional etiological factor in the development of BCC [3]. Indeed, scar tissue may have reduced immune surveillance capacity, enabling BCC cells to evade detection and proliferate within the fibrotic environment. The process is associated with immune privilege, preventing lymphocyte infiltration and interfering with the immune surveillance system [23]. The interaction between BCC cells and the surrounding stromal cells further influences tumor growth and invasion in scar tissue [24,25].

Diverse factors are implicated in the occurrence of BCC within scar tissue. These encompass chronic irritation, compromised cellular immunity, misplacement of epithelial cells, and mutations in tumor suppressor genes, all of which have been reported as etiological contributors to BCC formation in scar tissue [24]. Despite these reports, the exact underlying pathologic mechanism remains unclear, as highlighted in previous investigations. The research of Kennaway et al. [26] indicated that a compound emitted from human skin subjected to high temperatures can trigger the onset of cancer in the skin of mice. Additionally, an alternative hypothesis proposes that basal cells confined within a wound region contribute significantly to the progression of BCC during the epithelialization phase [27]. Other studies have provided insights into the influence of cytokines and growth factors on tumor initiation. Moreover, scientific investigations have demonstrated the presence of mutations in tumor suppressor genes within the context of skin cancers [28,29].

Ünverdi et al. [30] described three cases of patients displaying scarring resulting from prior burns and severe trauma. The wounds had healed via secondary intention, and BCC emerged within the scar tissue. These scar areas, located on the sun-exposed areas, were particularly susceptible to photodamage and chronic irritation. The process of secondary intention healing emerges as the most pivotal risk factor in scar tissue-related BCC. The loss of epithelial integrity creates a milieu of diminished vascularization, immunity, and reduces the number of collagen fibers, serving as a backdrop for chronic wounds within scar tissue. Phototoxicity, which incurs DNA damage and provokes the mutation of tumor suppressor genes is a plausible mechanism for BCC emergence within unstable, chronically irritated scar tissue.

The investigations conducted by Wong et al. [31] yielded noteworthy findings regarding the impact of wound-induced perturbations on the behavior of follicular stem cells carrying the SMO. In this context, these stem cells, originating from the follicular niche, exhibited a proclivity to migrate and subsequently give rise to tumors closely resembling BCC. Furthermore, Kasper and colleagues [32] made significant strides in elucidating the influence of the wound environment on the initiation frequency and growth of BCC-like lesions, particularly in a model featuring the homozygous inactivation of PTCH1. Lineage tracing shows that oncogene activation or wounding separately triggers keratinocyte emigration from the lower bulge and nonpermanent part of hair follicles towards the interfollicular epidermis. However, the combined effect of oncogene activation and a wound environment is necessary for these cells to participate in initiating BCC-like lesions at hair follicles’ openings and in the interfollicular epidermis.

**Figure 1:**
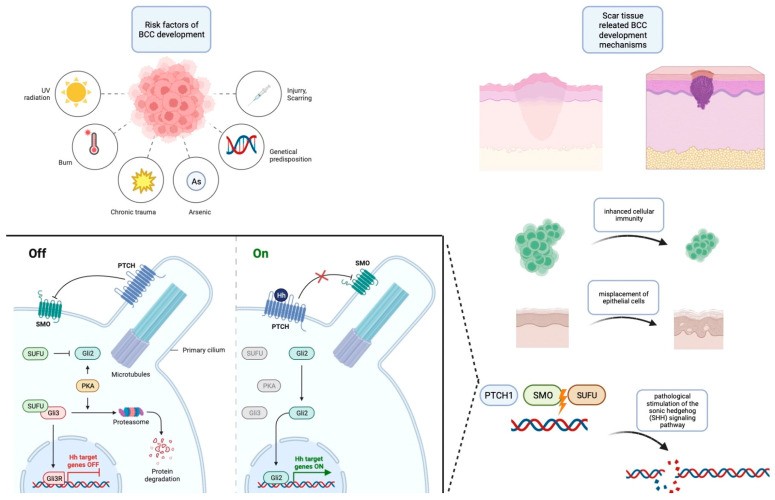
Risk factors for the development of BCC along with an outline of its pathogenesis. This figure was created with Biorender.com.

An essential dimension that emerges from these studies is the documented occurrence of acquired mutations in PTCH1 and SMO, which culminate in the overexpression of the SHH pathways, a hallmark of sporadic BCCs. Significantly, the evidence from these investigations underscores a pivotal insight: the synergy between genetic predisposition for BCC development and the presence of trauma and scar environments leads to an acceleration in the initiation of BCCs. Consequently, the current body of evidence collectively suggests that trauma can be posited as a potential risk factor contributing to the development of BCCs. The summarized risk factors and pathophysiology are contained within Figure 1.

Furthermore, the topic of significant concern and worthy of exploration is BCC arising within a post-vaccination scar; a pathomechanism which appears to interconnect all the previously mentioned elements. In our institution, two cases of BCC occurring in post-vaccination scar tissue were observed. The first one, described by Tyczyńska et al. [33], pertains to a 58-year-old woman, who developed nodular, eroded lesions, covered by eschars, approximately 52 years after a Bacillus Calmette–Guerin (BCG) vaccination. The second one, concerning a 60-year-old Caucasian man, is described in Table 1.

The clinical cases are not the sole instances documented in the literature. There have been limited reports of BCC occurrence in post-trauma scars. After these initial observations, a plethora of documented cases emerged, detailing instances where BCC manifested specifically at the sites of prior vaccination or burn scars. These documented cases often revealed a considerable temporal span, with some reports noting the development of BCC even after the passage of fifty years from the occurrence of the vaccination or traumatic event. Notable among these investigations is the work conducted by Noodleman and collaborators [34], who meticulously explored the history of trauma in 1774 patients diagnosed with BCC. Their findings demonstrated that 7.3% of these individuals had a documented history of trauma at the specific site where the BCC emerged. Additionally, Ozyazgan et al. [35] conducted a similar inquiry involving 92 patients and reported a history of prior trauma in 13% of these cases, further supporting the notion that trauma could be considered an etiological factor in the development of BCC.

Most post-vaccination cases allude to BCC development in BCG, smallpox, influenza, and travel vaccination scars. For instance, in 1980, Hendricks et al. [36] reported a case of BCC in a chickenpox vaccination scar in a 71-year-old patient. On physical examination, there was an indurated nodule with a central crust and peripheral telangiectasia on the right side of his chin. Hazelrigg et al. [37] described a 42-year-old female patient with erythematous, slightly scaly plaque surrounded by small pearly thread-like borders located on the post-smallpox vaccination scar in the left upper outer arm. The interval between vaccination and clinical presentation was 30 years. In 2004, Pace et al. [38] depicted a case of a 77-year-old man with a non-resolving ulcer in an influenza vaccination scar on the deltoid aspect of the upper arm, which occurred five months after vaccine injection. Moreover, Smith et al. [39] outlined a case of a patient, boosted against hepatitis A and typhoid, developing red, crusted plaque in his right arm’s deltoid region. These examples, along with others, demonstrate a common theme: scars, whether from vaccinations or other traumas, can act as loci for BCC due to altered cellular dynamics and reduced immune surveillance.

The cases of BCC developing specifically in BCG vaccination scars are particularly notable. The first encountered cases were reported by Ben-Hur et al. [40]. The study embraces a description of two patients; the first one, a 40-year-old male, was diagnosed with BCC five years before admission. The physical examination revealed an ulcerated, crusted vaccination site. The second one, a 36-year-old female claimed to have a minor, hyperkeratotic alteration in a vaccination scar, 13 years after BCG vaccine injection [40]. Furthermore, in 1979, Nielson et al. [41] reported a case of a 52-year-old female with a vividly detached, eczematous lesion stemming from BCG vaccination nine years before. Similarly, Braithwaite et al. [42] described a case of a 31-year-old woman who had perceived initial abnormalities in a vaccination site at the age of 25. On admission, examination disclosed a scaly, crusting lesion over the left deltoid region. Another two cases were reported by Smith et al. [39] in 2008. One of them was a 62-year-old female with an erythematous, scaly non-mitigating patch arising in a BCG booster scar, 13 years after the last of four BCG injections. In 2009, Polat et al. [43] highlighted the case of a 55-year-old male patient with a growing wound in a vaccination scar. Interestingly, in this case, the BCC had occurred in a patient with a Fitzpatrick type III skin classification that ruled out excessive exposure to UV light. In 2012 Kluger et al. [44] described a case of a 59-year-old male with an erythematous, scaly, sharply delimited alteration in a scar which stemmed from a tuberculosis vaccine given 40 years before. In the same year, Sari et al. [45] noticed an unevenly pigmented lesion located on a 53-year-old man’s right deltoid area. In the most recent report, Bostan et al. [46], in their study, reported a clinical example of a 59-year-old woman with a pinkish nodule with an ulcer in the BCG vaccine scar localized on the left upper arm.

Some assumptions can be made in studying the literature on the development of BCC in BCG vaccination scars. There have been 11 tumors reported, 6 in women and 5 in men, a ratio of 1.2:1. The average age of all patients is 51, and the age range is from 31 to 62. Moreover, no cases have been reported in childhood.

The prevalence of BCC in BCG scar is very seldom. Since reported lesions are miscellaneous, any alteration observed in the vaccination scar should raise suspicions of malignancy. The etiology of this phenomenon is yet to be elucidated. These clinical cases are not merely anecdotal but are indicative of a broader phenomenon where physical trauma and resulting scar tissue can predispose to BCC. This predisposition is likely due to factors such as chronic irritation or localized immune suppression. These elements are crucial in understanding the pathogenesis of BCC, as they highlight that skin carcinogenesis can arise not only from genetic mutations and UV exposure but also from specific local conditions.

## 4. Clinical Features of BCC

The typical clinical presentation of BCC is a plaque, nodule, or pink-hued tumor with a raised, pearly border or a translucent surface in the case of a nodule. Over time, the surface of the carcinoma may develop minor erosions, crusts, or ulcers. Clinical differential diagnosis of BCC encompasses several disease entities, such as actinic keratosis, Bowen’s disease, seborrheic keratosis, fungal skin infections, nummular eczema, psoriasis plaque, chronic discoid lupus erythematosus (DLE), scar tissue, lupus vulgaris, adnexal neoplasms originating from hair follicles, sebaceous glands, sweat glands, and amelanotic melanoma (the differential diagnosis is subsumed in Table 2).

Several subtypes of BCC are distinguished, like nodular, superficial or morpheaform. The most encountered subtype of BCC arising from a scar is the morpheaform subtype. Its clinical characteristics encompass pink-to-ivory-white, glossy, smooth, scar-like, indurated plaques or depressions displaying poorly defined borders. Frequently, there is concurrent atrophy, and in certain instances, telangiectasias, erosions, or small crusts may emerge. These lesions are renowned for their subtle presentation. Additionally recognized as infiltrating BCC, morpheaform BCC typically exhibits a higher proclivity for aggressiveness when compared to nodular and superficial BCC, as it has a tendency for subclinical spread with the potential to inflict extensive local tissue damage. Notwithstanding, BCC raised upon scar tissue is not apprehended as more aggressive [35].

## 5. Dermoscopy Findings

Dermoscopic examination unveils a rich array of vascular features that hold a prominent role in the diagnosis of BCC. Among these vascular structures, the arborizing vessels stand as a central hallmark within BCC lesions [72].

Turning to pigmented-related features, blue-gray ovoid nests emerge as well-defined, often confluent, pigmented ovoid or elongated areas. These nests contribute to the distinctiveness of BCC dermoscopy [73]. Another intriguing, pigmented feature, the maple leaf-like areas, showcases bulbous extensions connected at a base, typically exhibiting shades of brown or gray blue, ultimately forming a distinctive leaf-like pattern.

Additional pertinent dermoscopic elements include ulceration, which represents a shallow erosion of the epidermis penetrating the dermis, potentially covered by coagulated blood or serous crust [74]. White, red structureless areas denote diffuse dermal fibrosis or a fibrotic tumor stroma, appearing as regions of white to red color. Distinctly, white shiny streaks, known as chrysalis or crystalline structures, become visible through polarized dermoscopy, revealing orthogonal short and thick crossing lines, indicative of dermal fibrosis [72,74].

Intriguingly, the presence of multiple blue-gray dots and globules constitutes a prevalent feature, characterized by loosely arranged, well-circumscribed round to oval structures, generally smaller than blue-gray ovoid nests. In-focus dots within the lesion correspond to well-defined, small grey dots arranged in a loose manner, sharply focused [75]. Additionally, spoke-wheel areas, although rare in dermoscopy, hold high specificity for BCC, presenting as radial projections surrounding a central darker point, often displaying blue or grey hues [76]. This comprehensive repertoire of dermoscopic features contributes to the refined diagnosis and characterization of basal cell carcinoma, enhancing clinical insights and enabling improved patient management.

The dermatoscopic criteria for the morpheaform subtype of BCC, which represents the most prevalent subtype of basal cell carcinoma originating from scar tissue, encompass a more prevalent occurrence of ulceration. Additionally, it is noteworthy that linear branched vessels observed in mBCC generally exhibit finer characteristics, displaying a more scattered distribution with fewer ramifications compared to the arborizing vessels typically observed in the nodular subtype of basal cell carcinoma (nBCC) [73]. Moreover, an increased prevalence of pink-white structures is noted in this BCC subtype [66].

## 6. Histologic Features

The histopathological characteristics of BCC predominantly entail clusters of basal cells featuring small cytoplasm, prominent hyperchromatic nuclei, apoptotic cells, and an embedding within a fibromyxoid stroma. In paraffin-embedded sections, a phenomenon known as retraction artifact or clefting is often observed, creating a space between the tumor and the surrounding stroma. The occurrence of angiogenesis, the formation of new blood vessels, serves as an indicator of tumor progression and development. The stroma surrounding BCC exhibits an increased presence of microvessels, which correlates with local aggressive behavior [77,78].

BCC histopathological subtypes are categorized based on their proclivity for tumor recurrence. The subtypes with a lower risk of recurrence include nodular, superficial, pigmented, infundibulocystic, and fibroepithelial variants. Conversely, those with a higher risk of recurrence encompass micronodular, infiltrating, morpheaform, and basosquamous types [10,79,80]. Unfortunately, there is a lack of histological descriptions in the literature specifically addressing BCC development in scar tissue. Still, morpheaform basal cell carcinoma, which is the most common variant of BCC occurring within scar tissue, is characterized by the presence of tumor cell strands that are intricately embedded within a densely fibrous stroma [3]. The tumor cells are closely arranged in columnar formations, which can sometimes be as slender as one to two cells in width, all surrounded by collagen-rich fibrous stromal tissue. [81,82]

From clinical case reports, it is known that the histological examination of BCC developing on a scar post-BCG vaccination can reveal distinct features. Histological analysis may show an ulcerated multifocal BCC with a small granuloma in the subjacent dermis [42]. Another example includes histological findings of nests of basaloid cells with peripheral palisading and absent granulomatous infiltrate, confirming the diagnosis of nodular BCC [30]. Additionally, histology may reveal signs of collagenous scar tissue with retraction spaces and elastosis, along with atypical basal cells with peripheral palisading beneath the epidermis, consistent with a superficial BCC [31].

## 7. Treatment

BCC is the most common type of skin cancer, primarily caused by excessive exposure to ultraviolet (UV) radiation, especially from sunlight. While it typically does not metastasize, it can cause significant local destruction if left untreated. The management of BCC depends on various factors, including the clinical and histopathological characteristics of the lesion, its location, risk of recurrence, and the overall health of the patient. A practical classification of BCC distinguishes between “easy-to-treat” and “difficult-to-treat” types. Over 95% of BCCs are “easy-to-treat”, managed initially with standard surgery or alternative therapies. “Difficult-to-treat” BCCs include all locally advanced cases and typical BCCs that present specific management challenges. Of note is the importance of a multidisciplinary approach involving dermatologists, surgeons, clinical oncologists, and radiotherapists for the latter category, particularly in challenging anatomical regions like the eyelid or nose [2].

The surgery remains the undisputed gold standard for treating BCC (except for inoperable changes), regardless of the risk of potential recurrence. This method often represents the fastest and most effective approach, enabling a comprehensive histopathological diagnosis of the excised lesion. The National Comprehensive Cancer Network (NCCN) suggests using 4 mm clinical margins for low-risk tumors managed with standard excision alongside postoperative margin assessment (SEPMA) [2].

In the case of high-risk recurrent cancer, it is advisable, whenever feasible, to conduct an intraoperative assessment of the completeness of the procedure.

Mohs micrographic surgery (MMS) represents a specialized surgical approach employed for the extraction of locally invasive and high-risk skin cancers. The primary advantage of MMS lies in its capability to enable precise microscopic monitoring of the entire tumor perimeter while minimizing the removal of healthy tissue.

During the procedure, a narrow band of tissue is extracted around and below the clinical boundaries of the skin tumor. The specimen is typically removed with a 45-degree bevel to facilitate tissue processing. Swift freezing and sectioning of the tissue in a cryostat microtome allow for rapid tissue examination (approximately 15–30 min). Horizontal sectioning of the tissue enables comprehensive evaluation of nearly all tissue margins, both peripheral and deep, under the microscope. The process is repeated until the histopathologic margins of the tumor are confirmed to be negative [83]

Photodynamic therapy (PDT) is another option. It involves using photosensitizing agents activated by specific wavelengths of light to induce apoptosis in BCC cells. This is recommended for superficial and thin BCCs. Delving into the specifics, this technique involves the utilization of light (emitted from incandescent bulbs, LEDs, or lasers) to activate the photosensitizer applied to the diseased tissues, leading to the generation of cytotoxic, reactive singlet oxygen species. In this method, the photosensitizer employed is d-aminolevulinic acid (ALA) or its methyl derivative, methyl aminolevulinate (MAL) [84]

Radiotherapy can be considered for elderly patients, especially those who cannot undergo surgery due to medical comorbidities, with BCC located on the face. However, it should be noted that while radiotherapy is a viable option, its recurrence rates and cosmetic outcomes may be less favorable compared to surgery. Moreover, radiation therapy demonstrates slightly higher efficacy in primary BCC cases than in recurrent cases, as well as higher effectiveness in lesions of smaller diameter and nodular subtypes. Additionally, radiation therapy plays a crucial role as an adjuvant treatment method for patients following non-radical excision or tumor cytoreduction, where the initial procedure remains incomplete due to tumor infiltration of critical structures, as well as post-lymphadenectomy for regional lymph node metastases [85,86]

Cryosurgery is an ablative technique involving the application of liquid nitrogen or nitrous oxide to induce thermal necrosis of tumor cells by generating low temperatures (in the range of −50 °C to −60 °C) at the base of the tumor tissue. This method finds application in the treatment of superficial types of low-risk BCC, with sizes up to 2 cm. Cryosurgery is not recommended for treating large tumors, aggressive histologic subtypes, cases where the tumor is fixed to the underlying bone, recurrent tumors, or instances involving deep penetration. To ensure maximal surgical extirpation, the tumor tissue along with a margin of healthy skin should be destroyed. Wound healing after the procedure generally progresses uneventfully, resulting in the formation of small, cosmetically favorable scars. Consequently, its use is discouraged in areas with hair to prevent scarring alopecia and in the lower legs to prevent ulceration [87,88]

Oral hedgehog (HH) inhibitors have shown promise for the treatment of locally advanced and metastatic BCCs. Vismodegib and sonidegib, small oral targeted Smo inhibitors, have been approved for first-line treatment. These inhibitors have demonstrated significant efficacy, with objective response rates for locally advanced BCC ranging from 48.5% to 60.3% for vismodegib and 46.5% for sonidegib. However, it is essential to be aware of potential side effects leading to treatment discontinuation.

While HH inhibitors offer a promising treatment option, the possibility of developing resistance necessitates ongoing research into identifying resistance biomarkers and active drugs for second-line treatment [89]. Additionally, a recent review suggests exploring the use of sonidegib as a neoadjuvant therapy in combination with surgery. This approach could potentially reduce the tumor size, making it eligible for less extensive surgical excision, thereby preserving function and aesthetic appearance [90,91].

Immunotherapy with anti-PD1 checkpoint inhibitors like cemiplimab is being explored as a potential treatment option for locally advanced/metastatic BCC in patients intolerant to previous therapy or experiencing stable disease. These inhibitors have shown promise, with objective response rates observed in some patients, highlighting the importance of ongoing research in this area [92].

In summary, the treatment of BCC depends on the type of lesion, its location, and the overall health of the patient. A multidisciplinary approach, including surgery, topical therapies, radiotherapy, and targeted agents, is essential for managing both “easy-to-treat” and “difficult-to-treat” BCCs, ensuring optimal outcomes, recurrence prevention, and the preservation of organ function in challenging anatomical regions.

## Figures and Tables

**Figure 2:**
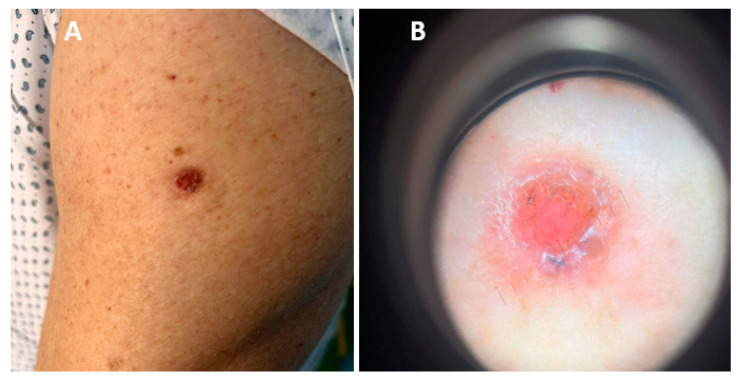
(**A**) Ulcerative nodule on the left upper arm localized upon BCG vaccination scar. (**B**) Dermoscopic picture: pinkish, non-structural area with no visible vessels, few yellowish and dark grey globules, scales and positive “sticky fiber” signs.

**Figure 3:**
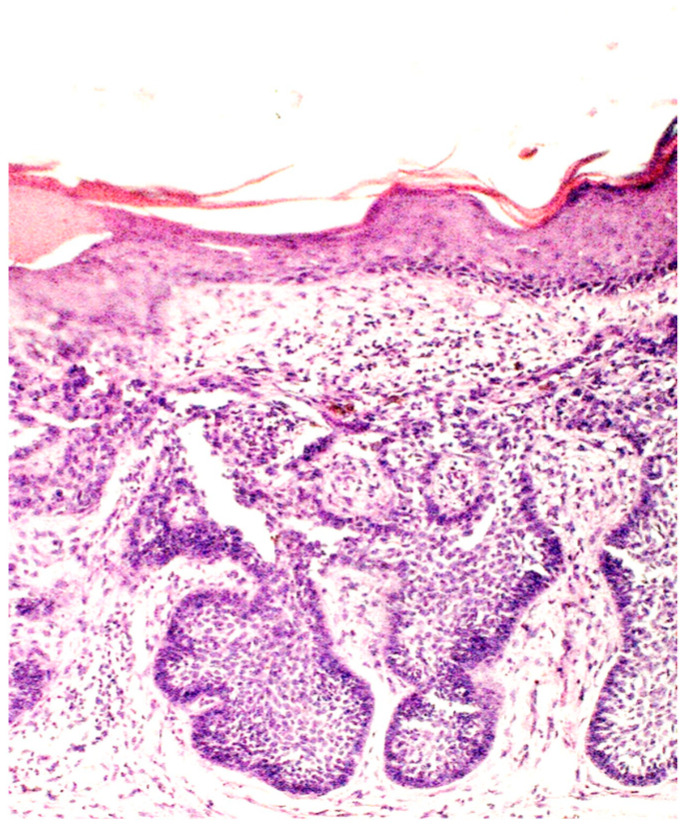
Basaloid nest accompanied by collagenous scar tissue and elastosis (H&E; original magnification ×100).

**Table 1:** Clinical case of patient presentation, diagnostic findings, and treatment outcomes.

Clinical presentation	A 60-year-old Caucasian man was referred to the dermatosurgical unit for a diagnosis and surgical treatment of a nodular lesion developing in a BCC scar on his left arm. The lesion initially occurred two years before admission. The patient was vaccinated in childhood pursuant to the Polish recommended schedule. He had no history of radiation, excessive sun exposure or previous skin cancer.
Workup	The physical examination revealed an eroded nodule covered by crusts (Figure 2A). The dermoscopy revealed a central pinkish non-structural area with no visible vessels, few yellowish and dark grey globules, scales and positive “sticky fiber” signs (Figure 2B).
Treatment and course	The lesion was surgically removed, and the subsequent histological examination revealed basaloid nests, elastosis and collagenous scar tissue. Histological and clinical correlation was consistent with a diagnosis of BCC occurring in a BCG scar (Figure 3).

**Table 2:** A diagnostic differentiation of basal cell carcinoma (BCC) based on clinical, dermoscopic, and histological features.

Differential Diagnosis of BCC in Scar and Other Adnexal Neoplasms	Clinical Features	Dermoscopic Features	Histological Features	Source
BCC	Gradual growth translucent or pearly nodule primarily on sun-exposed regions of the skin frequently exhibiting telangiectasia, prone to ulceration or crusting translucent or pearly nodule	Blue-gray ovoid nests leaf-like areas with serpentine vessels central ulceration or erosion	Lobules of basaloid cells palisading of nuclei peripheral palisading increased mitotic activity. Tumor cells infiltrating scar tissue	[47]
Hypertrophic scar	Raised or flat, erythematous, and firm lesion limited to the confines of the initial wound	Homogeneous pinkish or reddish color lack of vascular structures	Elevated quantity of fibroblasts heightened concentration of collagen fibers within the dermal layer	[48,49]
Keloid	Purplish red firm, smooth, and raised, extending beyond the confines of the original injury	Homogeneous pinkish or reddish color lack of vascular structures	Spiral patterns and clusters of notably thick and uniform collagen bundles (dense fibrils), irregularly situated within the dermal region (known as keloidal collagen)	[48,49]
SCC arising in a scar	Gradually increasing, solid, skin-toned to red plaques or nodules exhibiting significant hyperkeratosis either ulceration or exophytic and infiltrative growth patterns	Keratinization, leading to prevalent white areas without distinct structures. Irregular clusters of white circles around hair follicles blood vessels exhibit diverse shapes, ranging from irregular round or coiled to looped, serpentine, branched, or showing polymorphic morphology	Clusters of squamous epithelial cells originate from the epidermis and progress into the dermal layers. Malignant cells typically exhibit large size, abundant eosinophilic cytoplasm, and frequently possess a sizable, often vesicle-filled nucleus. Variable forms of keratinization, such as the presence of keratin pearls	[50,51]
Dermatofibrosarcoma protuberans (DFSP)	Asymptomatic, infiltrative plaque with a violet, red-blue, or brownish hue and firm consistency exhibiting gradual growth, attaining dimensions of several centimeters in diameter	Pigmented reticular pattern. Unstructured regions displaying a light brown hue, distinct bright white streaks. Background coloration with a pinkish tone, and areas lacking structure, manifesting as either hypo- or depigmented	Spindle cells arranged in a storiform pattern. No palisading or mitotic activity infiltrative growth in the dermis	[52,53]
Merkel cell carcinoma (MCC)	Swift proliferation, absence of symptoms a firm consistency, a reddish-violet irregular nodule most commonly occurring in immune-suppressed individuals, older than 50 years of old, in UV-exposed fair skin	Milky red areas often coincide with linear, irregular vessels, red dots, a varied vascular pattern. the absence of pigmentation, blue-gray veiling, and the presence of white, structureless areas described as shiny or non-shiny streaks and globules	Composed of densely packed blue-hued cells typically centered within the dermal layer, it frequently extends into the overlying epidermis. often takes the form of sheets, occasionally nests, and seldom ribbons. lymphovascular invasion tends to be a common characteristic	[54,55]
Amelanotic melanoma	Asymmetrical macules, potentially displaying a uniform pink or red hue with a subtle light tan, brown, or grey pigmentation at their edges. The borders of these lesions might be either well-defined or unclear	Diverse range of vascular patterns—serpentine vessels, irregular linear vessels, pinpoint (dotted) vessels, and hairpin vessels concurrent presence of dotted and irregular linear vessels serves as a positive diagnostic indicator for amelanotic melanoma	Atypical melanocytes without pigment epithelioid or spindle cell morphology may have mitotic figures and nucleoli	[56]
Microcystic adnexal carcinoma (MAC)	Slow-growing, smooth, flesh-colored, or yellow-hued nodule found typically on the face or neck. perineural spread can occur in some cases, leading patients to report sensations like numbness, tingling, pain, burning, and itching	White, structureless areas with fine arborizing telangiectasias	Small basaloid cells or keratocysts primarily located at the surface, contributing to the term “microcystic”. Clusters and threads of basaloid epithelial cells showing duct-like structures, invading both the dermal and subdermal layers.	[57,58]
Trichoblastoma	Solitary, slow-growing nodule, often skin-colored or slightly pigmented and ulcerated; typically located on the scalp or neck	Non-specific; may present with blue-gray ovoid nests and white streaks; absence of arborizing vessels	Scant or absent mitotic activity; minimal or absent necrosis, lack of inflammatory infiltrate. absence of connection with the epidermis. myxoid stroma induction	[59,60]
Trichoepithelioma	Multiple, small, skin-colored papules; predominantly on the face; occasionally familial	Absence of large arborizing vessels; may show milia-like cysts and white streaks; less vascular features than BCC	Similar to trichoblastoma but often with horn cysts, calcifications, and more prominent follicular differentiation	[61,62]
Basaloid follicular hamartoma	Cysts, plaques or small skin-colored or pearly-translucent papules	Light brown pigmentation, and subtle blue-gray dots; typically lacks the ulceration and associated vascular structures seen in BCC	Anastomosing strands and cords of basaloid cells with hyperchromatic nuclei and scant cytoplasm; rare mitotic figures; minimal stroma; normal dermis between affected follicles	[63,64]
Pilomatricoma	Firm, solitary, and often deeply seated nodules; typically in children, commonly on the head and neck. Often exhibits the “tent sign” (stretched skin forming tent-like structure) and “teeter-totter sign” (pressing one edge causes the opposite edge to protrude)	White and yellow opaque areas, blue-gray granules, and peripheral vascular network; presence of “chalky” white structures	Well-demarcated lesion with basaloid and shadow cells, calcification, and ossification; can show variable keratinization	[65,66]
Sebaceous adenoma	Yellowish, lobulated papules or nodules, commonly on the face; may be associated with Muir–Torre syndrome	Loosely arranged yellow comedo-like globules and branching arborizing vessels (less vascular than BCC)	Lobulated architecture with mature sebaceous differentiation, basaloid germinative cells, and occasional mitotic figures	[67,68]
Eccrine poroma	Skin-colored, pink-red, or occasionally blue-black, solitary, firm papule, plaque, or nodule, sometimes with a rim. Smooth, shiny, scaly, verrucous, or papillomatous surface secondary ulceration and erosion	Vascular pattern with branching vessels, white streaks, and a polymorphous vascular pattern; may mimic BCC with arborizing vessels; however, the vascular structures appear less in focus.	Dermal nests of poroid cells with ductal differentiation; often showing a connection to the epidermis.	[66,69]
Actinic keratosis (AK)	Solitary, poorly defined, reddish macules or papules, characterized by a course scale. Often indistinct, potentially easier to feel while palpating than to visually discern	“Strawberry pattern”, featuring an erythematous vessel pseudo-network, noticeable follicular openings, and a white halo surrounding the lesion	Acanthosis, parakeratosis, dysplastic keratinocytes, varying levels of dermal soral elastosis	[70,71]

## Data Availability

No new data were created or analyzed in this study. Data sharing is not applicable to this article.
